# Development, implementation, and testing of LGBTQIA+ care curriculum for health science professionals: Research protocol.

**DOI:** 10.12688/f1000research.140518.1

**Published:** 2023-11-06

**Authors:** Mamatha Shivananda Pai, Renjulal Yesodharan, Vikram Palimar, Latha Thimmappa, Bhavana B. Bhat, Nirmal Krishnan M., Deeksha Shetty, Bontha V. Babu

**Affiliations:** 1 Department of Child Health Nursing, Manipal College of Nursing, Manipal Academy of Higher Education, Manipal, Karnataka, 576104, India; 2 Department of Psychiatric Nursing, Manipal College of Nursing, Manipal Academy of Higher Education, Manipal, Karnataka, 576104, India; 3 Department of Forensic Medicine and Toxicology, Kasturba Medical College, Manipal Academy of Higher Education, Manipal, Karnataka, 576104, India; 4 College of Nursing, All India Institute of Medical Sciences, Kalyani, West Bengal, 741245, India; 5 Department of Pharmaceutical Regulatory Affairs and Management, Manipal College of Pharmaceutical Sciences, Manipal Academy of Higher Education, Manipal, Karnataka, 576104, India; 6 Manipal College of Nursing, Manipal Academy of Higher Education, Manipal, Karnataka, 576104, India; 7 Division of Socio-behavioural, Health Systems & Implementation Research (SHI), Indian Council of Medical Research, New Delhi, Delhi, 110029, India

**Keywords:** Curriculum, Faculty, Health Sciences, Health professional, India, LGBTQIA+, Research Protocol, Students, SDG 3, SDG 10

## Abstract

Lesbian, Gay, Bisexual, Transgender, Queer, Intersex and Asexual (LGBTQIA+) people struggle to identify a healthcare service that understands their problems and needs. Additionally, healthcare professionals also find it difficult to care for LGBTQIA+ as very little is studied or heard about management. The article presents a protocol for a pilot study aimed at the development of an LGBTQIA+ care curriculum for health science professionals. The study includes Phase I: The development of a curriculum based on a literature review and focus group discussion among LGBTQIA+ individuals, and Phase II: Pilot testing of LGBTQIA+ care curriculum. The study outcome will reflect the improvement in the knowledge of healthcare professionals on LGBTQIA+ care.

## Background

The Lesbian, Gay, Bisexual, Transgender, Queer, Intersex, and Asexual (LGBTQIA+), are a varied group of people with different gender identities, sexual orientations, and reproductive development. All members of the LGBTQIA+ community have different healthcare-related problems and requirements, even though they are sometimes grouped as a coalition. However, they share the stigma and discrimination that have hindered them from accessing quality healthcare in several ways ( Agarwal & Thiyam, 2022; MacKenzie *et al.*, 2020). Human health and social systems are interrelated. According to a Yang (2021)’s study, LGBTQIA+ individuals confront social challenges such as stigma, homophobia, discrimination, coming out, insufficient social support systems, and a lack of LGBTQIA+-friendly medical resources that not only put them in danger for physical and mental harm but also put their health at risk. LGBTQIA+ individuals face discrimination, violence, and bullying as they come to terms with their identity. LGBTQIA+ individuals are more vulnerable to mental health problems including anxiety and depression as a result of this type of minority stress ( Yang, 2021).

Individuals can self-identify in different ways, including gender identity and sexual orientation. For the remainder of the text, we will refer to this spectrum collectively as the LGBTQIA+ community. The terms “L” for lesbian and “G” for gay refer to people who are attracted to people of the same gender; “B” for bisexual means they are attracted to people of both genders; and “T” for transgender means they identify as a gender other than the one they were given at birth. The ‘+’ recognizes and includes additional identities and orientations not covered by the initials and refers to the community’s dynamic nature ( Agarwal & Thiyam, 2022). A person’s sexual and emotional attraction to another person, any resulting behaviour, and/or social connection are all considered to be part of their sexual orientation. The strongly held, innate notion that a person has of being a boy, a man, or another member of the male, female, or alternate gender is considered their gender identity. The way society views homosexuality varies widely between countries and historical eras. Heterosexuality is now accepted as the standard across the globe. LGBTQIA+ individuals face stigma and stereotypes in many countries. Although the Delhi High Court decriminalized homosexuality in 2009, the Indian Supreme Court affirmed section 377 of the Indian Penal Code, which makes adult consenting same-sex contact a crime, on December 11, 2013 ( Kar *et al.*, 2018). The Supreme Court of India recognized LGBTQIA+ people as the third gender in April 2014, and any discrimination against them was viewed as a violation of their constitutional rights.

Despite recent developments in the acceptance of LGBTQIA+ individuals, education on LGBTQIA+ health requirements for health professionals still lags far behind despite evidence showing a tremendous rise in LGBTQIA+ acceptance and the achievement of equality in many sectors. Multiple studies and reviews of health issues have shown a persistent gap in healthcare education, with no standard texts that include information concerning care for LGBTQIA+ individuals ( Keuroghlian *et al.*, 2017). LGBTQIA+ community members encounter several difficulties regarding sexual orientation and gender identity in a heteronormative culture. Additionally, they frequently experience prejudice and sexual assault. Further, their medical requirements are more likely to be overlooked or socially rejected due to their sexual orientation and gender identity, which can affect their medical rights and the medical care they receive. LGBTQIA+ individuals frequently struggle with coming out when dealing with medical professionals. Two things worry them; First, medical professionals’ ignorance, bias, and discrimination may impair their right to get medical care. Second, incomplete information disclosure may influence a disease’s diagnosis or possibly lead to a misdiagnosis. LGBTQIA+ individuals must carefully balance the risks of coming out with the benefit of having the right medical attention and support. These factors frequently lead to psychological pressure, which is harmful to both physical and mental health ( Yang, 2021).

The stigmatized and discriminated populations must be addressed by healthcare practitioners. Understanding how medical students feel about homosexuality is crucial for improving the healthcare system. Patients who identify as LGBTQIA+ have encountered stigmatization, discrimination, and even refusal of care within the healthcare system ( Kar *et al.*, 2018). In 2017, the Joint United Nations Programme on HIV/AIDS UNAIDS report stated that LGBTQIA+ individuals made up 4.3% of the population in India who were at high risk of contracting AIDS ( Kar *et al.*, 2018). Due to the underrepresentation of such information in medical school curricula, clinicians may not be aware of or sensitive to the needs and issues faced by LGBTQIA+ patients when they encounter them ( Sequeira *et al.*, 2012; Magnus & Lundin, 2016).

Healthcare professionals are frequently not trained in or sensitive to the requirements of LGBTQIA+ individuals’ health. Additionally, it might be challenging for professionals to talk about identity in general, especially when it comes to sexual orientation and gender identity. Medical education institutions can sometimes become the breeding ground for a heteronormative ideology that supports heterosexualism ( Lundin, 2011; Magnus & Lundin, 2016). Heteronormativity refers to the belief that only two opposite and mutually complementary genders exist – or that gender and sexual variation simply does not exist in social institutions ( Kannisto, 2019). Due to a lack of specialized expertise and/or heterosexist attitudes on the part of healthcare personnel, LGBTQIA+ people face barriers to accessing sufficient healthcare in this way. The heterosexist attitudes may result in inaccurate risk assessments for sexually transmitted infections (STIs), pregnancy, and the inadequate or incorrect use of screening equipment. These obstacles may negatively affect the treatment management and, ultimately, the health of these people ( Wahlen *et al.*, 2020).

The LGBTQIA+ community is substantially more likely to experience various risk factors for poor health than heterosexual people, such as being less likely to have health insurance, being more likely to be obese, smoking more regularly, and engaging in binge and heavy drinking, the population’s age warrants additional attention. In addition, compared to heterosexual women, lesbian and bisexual women may undergo fewer preventative screenings for colon, breast, and cervical cancer. This is partly due to fear of not receiving respectful healthcare. Healthcare professionals are at the forefront of this effort and have the chance to treat everyone with respect, regardless of their sexual orientation and gender identity. Lack of time, finances, education, and clinical experience are a few obstacles that can prohibit physicians from providing respectful medical care ( Walker *et al.*, 2016). Doctor-patient interaction is essential for enhancing people’s health ( Parker & Bhugra, 2000). Patients may opt to keep their sexual orientation and gender identity private. In such situations, healthcare professionals must be vigilant and compassionate to deliver the best care ( Grabovac *et al.*, 2014).

There are no such policies or curricula for treating LGBTQIA+ patients in India’s healthcare sector. LGBTQIA+ people have common social tendencies and decisions that impact their behavior while seeking healthcare, preventative health measures, and illness risk ( Kaufman *et al.*, 2014). It is vital to make accessible, responsive, appropriate, and well-resourced healthcare services provided by knowledgeable and trained healthcare professionals to support a better patient experience. Higher education institutions and healthcare organizations have a significant role in developing curricula ( Cui, 2023) that can be accessed by all groups, including those who identify as LGBTQIA+ ( McCann & Brown, 2018). Health institutions need to make room for individuals from different sexual orientations and gender identities and communicate a sense of welcome ( Hafford-Letchfield *et al.*, 2017).

To better prepare medical students to treat these underserved areas and lessen healthcare inequities, medical teachers play a crucial role ( Alhanachi *et al.*, 2021; Chinchilla & Arcaya, 2017). Training medical students during their studies can help them feel more at ease when caring for these patients and give better treatment, an essential technique for improving understanding and attitudes about LGBTQIA+ persons among physicians ( Wahlen *et al.*, 2020). Inculcating positive LGBTQIA+ attitudes among medical professionals plays a great role in reducing homophobia and transphobia ( Gegenfurtner, 2021). The scope of the proposed research is to develop a curriculum on the care of LGBTQIA+ and pilot test on faculty and students of health sciences in the form of workshops. For this purpose, a need assessment through a review of the literature and focus group discussion with LGBTQIA+ individuals will be done.

## Research plan

### Research questions

How is an LGBTQIA+ curriculum effective on the knowledge of healthcare providers in caring for LGBTQIA+ individuals?

***Primary research question***

What are the healthcare needs of LGBTQIA+ people?

To answer the primary research question, lead questions will be used to assess LGBTQIA+ individuals’ care needs, barriers to accessing care, and expectations from healthcare providers.

***Secondary research question***

What is the knowledge of health science students and faculty on LGBTQIA+ health needs?

To achieve this question, a structured knowledge-based questionnaire on LGBTQIA+ care will be administered to the health science students and faculty to assess their knowledge about LGBTQIA+ care.

***Hypothesis/Assumptions***

It is hypothesized that a curriculum on LGBTQIA+ care will significantly increase the knowledge of the healthcare professional in the care of LGBTQIA+ individuals.

Based on the previously published literature related to the needs of LGBTQIA+; it is assumed that

a. LGBTQIA+ individuals experience stigma and discrimination.
b. The present health science curriculum does not have a specific curriculum that deals with the management of LGBTQIA+ health problems.
c. There is a lack of structured guidelines for managing the health conditions of LGBTQIA+ people.

***Design***

**Figure 1.**
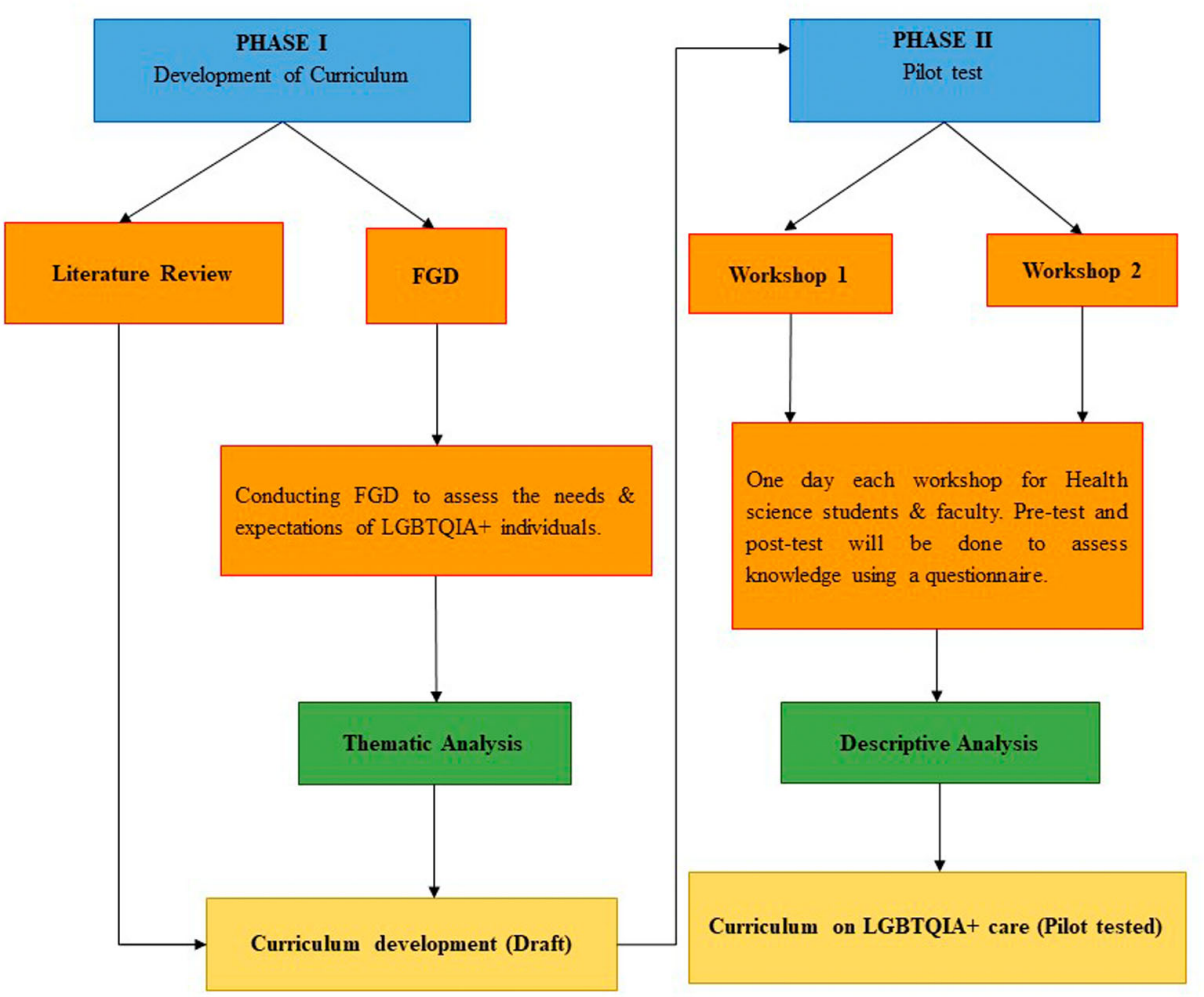
A conceptual framework of the research design.

The study includes two phases: Phase I - development of the curriculum based on the literature and the need assessment through the focus group discussion among LGBTQIA+ individuals; and Phase II - A pilot testing of the curriculum for health science faculty and students with pre- and post-test assessment design. A conceptual framework of the research design is shown in Figure 1.

### Phase I: Developing the curriculum

**a. Literature review**

The LGBTQIA+ curriculum for health sciences will be developed based on the detailed literature review and analysis of focus group discussion (FGD). The literature will be reviewed in detail through different sources to understand the needs of the LGBTQIA+ community. This includes academic research databases such as SCOPUS, Web of Science, PubMed, Science Direct, and CINAHL Complete. The grey literature search will also be initiated. The literature search is primarily conducted to find out the existing health care needs of the LGBTQIA+ community and it does not involve the steps of systematic reviews. The literature on the needs, problems, and expectations of LGBTQIA+ will be studied and used for developing the curriculum.

**b. Focus group discussion**

The FGD will be conducted in a district government office in coordination with the health department of the local government. The Principal Investigator will moderate the FGD by following the standard methodology ( Kitzinger, 1995). The data will be collected from the participants after obtaining administrative permission from the authorities. The participant information will be explained in the local language, and a signed informed consent will be taken from the participants for collecting the data and for audio recording. The sociodemographic data of the participants will be taken, and the participants will self-report their sexual orientation and gender identity. The lead questions prepared for the FGD will be used for conducting the discussion ( Box 1). After receiving consent from the participants, a discussion will be initiated using the lead questions. Participants are encouraged to communicate and discuss their healthcare needs and expectations from the healthcare facility. Based on the participant’s response, probe questions will be asked till all the questions are answered. The FGD will be recorded using an audio recorder with the consent of the participants, and nonverbal communication during the discussion. It will be used as an adjunct while transcribing the discussion. A sociogram also will be drawn to record the interactions among the participants. The FGD session will be closed by the moderator after summarizing the important points to the participants. The team will thank the participants and compensate them for the quality time spent by the participants. The data gathered will be thematically analyzed ( Braun & Clarke, 2021; Kyngäs *et al.*, 2019), and will be used for developing the curriculum for health science students.

Box 1. **FGD lead questions.**

a. Describe your experience of visiting a hospital/clinic. (Probe-interaction with health professionals)

b. What are your health needs that require medical attention?

c. Explain the changes you expect in addressing your health needs.

d. What are your expectations in addressing you?

e. What are your expectations from the health care provider when you visit the hospital/clinic?

f. How can a healthcare provider best assess your health needs?

g. How can healthcare providers help you in the treatment of any illness?

h. What are the challenges you face in seeking treatment for your health needs?

i. Explain any other information you would like to bring to our attention.

**c. Development of LGBTQIA+ care curriculum**

Based on the literature review and FGD analysis, the authors will draft the LGBTQIA+ Care curriculum. The draft will be sent to experts for content validation, and the final curriculum will be developed based on the expert’s opinions. The developed curriculum for the care of LGBTQIA+ individuals will be used for Phase II.

### Phase II: Pilot testing of the curriculum

The curriculum will be pilot-tested in the form of workshops for faculty and students of health sciences in the workplace. The faculty and students will be from nursing, medicine, psychology, and nurse educators. The workshop will be for one day. Knowledge will be assessed before and after the workshop using a questionnaire. The tentative topics covered in the workshop are, introduction to LGBTQIA+ care, inclusive communication, LGBTQIA+ community and health care- treatment management, mental health services for LGBTQIA+, research and evidence-based practice, interpersonal communication, ethical and legal issues in LGBTQIA+ health care. Feedback from the participants will be also taken for the modification of the curriculum. The curriculum will be finalized after the pilot study, and recommendations will be submitted to health sciences institutions, their regulatory bodies, and funding agencies.

### Participants

*Phase I*

Phase I includes an FGD to assess the healthcare needs among self-identified LGBTQIA+ individuals. The result of the FGD and the literature review will help in preparing the curriculum.

One face-to-face FGD of 10-15 individuals who are a representative number of the self-reported LGBTQIA+ community will be recruited using purposive sampling. The details of the participants will be collected from the district authorities. They will be contacted to schedule the FGD. The sample size is kept under 10-15 to support the depth of FGD and subsequent analysis.

**Inclusion criteria**

• The participants who self-report as LGBTQIA+ community and can speak in Kannada or English.
• Individuals who are above the age of 18 years.
• Individuals who are willing to participate in the focus group discussion.

**Exclusion criteria**

• Any person who is not belonging to the LGBTQIA+ community.
• Any individuals who are crossdressers.
• Any member of the LGBTQIA+ community who has a diagnosed mental disorder listed under chapter F of ICD 10, except gender identity disorder.

**Drop-out criteria and withdrawal**

Data collection from participants will be ceased when they withdraw their consent to participate, and the concerned data will be excluded during analysis.

### Phase II

The participants for phase II will be selected from the health sciences institutions offering medical, nursing, and other health science programs through their heads of the institution. Faculty and students will be recruited face to face to the study, and the curriculum will be pilot-tested both among the faculty and students through workshops. No dropouts are expected as the workshop is for one day.

The number of participants for this group will be based on the formula:

$$N=\left(\mathrm{Z\alpha}+\mathrm{Z\beta}\right)2/\left(E/S\Delta \right)2$$

where;

α (two-tailed) = % Threshold probability for rejecting the null hypothesis. Type I error rate.

β = % Probability of failing to reject the null hypothesis under the alternative hypothesis. Type II error rate.

E = Effect size

S _Δ_ = Standard Deviation of the change in the outcome

*Zα* = Standard normal deviate for *α* = 1.9600

*Zβ* = Standard normal deviate for *β* = 0.8416

*B* = ( *Zα* + *Zβ*)2 = 7.8489

*C* = ( *E*/ *S*Δ)2 = 0.2844

*N* = *B*/ *C* =27.5937

The *N* thus calculated is rounded up to 30 participants from students and faculty groups.

Students from medical (n = 10), nursing (n = 10), clinical psychology (n = 5), and senior nurses from the teaching hospital (n = 5) will be trained in the ‘student workshop’ whereas faculty from medicine (n = 10), nursing (n = 10) and psychology (n = 5) and middle-level nurse managers or educators (n = 5) will be trained in the ‘faculty workshop’. Undergraduate students and faculty are excluded from the ‘student workshop’, and similarly, students are excluded from the ‘faculty workshop’.

### Outcome measures

The outcome variables will be:

Phase I:

• Needs of the LGBTQIA+ individuals (analyzed) from FGD
• Curriculum on LGBTQIA+ for the health professionals

Phase II:

• Knowledge of health professionals on the care of LGBTQIA+ individuals.

***Primary outcome***

The healthcare needs of LGBTQIA+ individuals will be assessed, and the data gathered will be used in developing the curriculum for health science students.

Secondary outcome

The curriculum developed using the data gathered by FGD, literature review, and experts’ inputs will positively influence how LGBTQIA+ individuals are getting cared for by healthcare providers.

**Plan for data analysis**

**Phase I**

The FGD will be analysed by thematic analysis ( Braun & Clarke, 2021; Renjith *et al.*, 2021). The focus of the FGD analysis is not to identify the individual contributions to the discussion but to present the spectrum of opinions of the entire group ( Van Eeuwijk & Angehrn, 2017). The data will be divided into simpler text units for coding and the coding will be done manually. Units of meaningful text corresponding to similar codes will be grouped and categorized systematically by the authors. Any differences in the process of coding and categorizing will be resolved by discussion among the authors. Consensus will be achieved during these face-to-face discussions. The codes will be categorized into inductive and deductive and inductive codes will be content-driven and raised by participants, whereas deductive codes originated from the discussion guide and will then be verified with data ( Hennink *et al.*, 2019).

**Phase II:** Descriptive statistics like frequency and percentage will be used for the data analysis of the workshop participants. The knowledge data will be analysed using a paired t-test.

**Dissemination**

Results will be disseminated via presentations at appropriate scientific conferences and meetings of professional bodies. The study will also be published in peer-reviewed journals, professional and institutional repositories, etc. The results will be discussed with governmental bodies and other stakeholders for broader implementation.

**Status of the study**

The study team completed the draft curriculum and is currently in the process of pilot testing the LGBTQIA+ care curriculum (phase II). The study is expected to be completed by December 2024, and the results will be published by 2024 and 2025. This protocol will help in reducing unnecessary duplication of effort and the costs of future studies.

## Discussion

The previous study findings show that LGBTQIA+ individuals face more difficulties accessing care because of their sexual orientation and gender identities. The study found that young adult lesbians had a harder time getting access to care than young adult homosexual males. This finding is consistent with earlier research that identified differences in the healthcare experiences of sexual minority individuals. Additionally, due to negative experiences associated with their sexual orientation and identity, gay males were more prone than lesbians to delay care. Young men may be more forthcoming about their sexual orientation and identity in healthcare, which may increase the potential for negative experiences, whereas young women may be more reticent to disclose their sexual orientation and identity to providers and as a result feel limited in their ability to access affirming care ( Macapagal *et al.*, 2016).

Contrary to earlier findings, few individuals claimed to have encountered LGBTQIA+-related discrimination in medical settings. Furthermore, the majority stated that telling their provider about their gender identity and sexual orientation had a neutral to a positive impact on their care ( Mosack *et al.*, 2013). The findings from previous studies suggested that changes in the healthcare system will promote inclusive care. Studies have shown that reluctance to talk about sexual orientation and gender identity was brought on by ignorance of the medical requirements for LGBTQIA+ patients ( LaVaccare *et al.*, 2018). According to a previous research study, students who had more contact with LGBTQIA+ patients were more likely to ask about a patient’s sexual orientation and gender identity and check for children in the patient’s family. The disclosure of this information during patient interactions may be improved by early intervention by educators who teach students appropriate questions to ask during the history-taking process ( Sanchez *et al.*, 2006). The current study will explore the healthcare needs of LGBTQIA+ and will result in education programs and initiatives that can improve knowledge about LGBTQIA+ individuals and will provide practical techniques that can easily be included in the health science curriculum which will help in reducing the disparities. The curriculum will improve the healthcare professionals’ knowledge about sexual orientation, gender identity, sexual behaviour, and sex anatomy comfort of LGBTQIA+ patients. It will also enhance the self-confidence and comfort of the healthcare professionals to treat LGBTQIA+ people. The curriculum will summarize the healthcare needs, barriers, and expectations based on FGD and primary care recommendations for LGBTQIA+ patients. This study will support the necessity for a curricular framework to reduce unconscious bias among students of healthcare professions toward LGBTQIA+ patients.

## Conclusion

The study will address the critical gap in the medical curriculum about LGBTQIA+ care. The curriculum will enhance the skills and knowledge of the healthcare providers about the needs and expectations of LGBTQIA+ individuals. It will promote healthcare professionals’ positive attitudes toward LGBTQIA+ patients and improve comfort working with LGBTQIA+ patients. The curriculum will guide researchers and educators looking to reduce prejudice against LGBTQIA+ patients in healthcare professionals, as well as a framework for teaching students to recognize and overcome their own biases. Educational Strategies that reduce bias in healthcare providers are essential steps to improving LGBTQIA+ communities’ access to treatment and reducing health inequalities.

### Project plan

**Figure 2.**
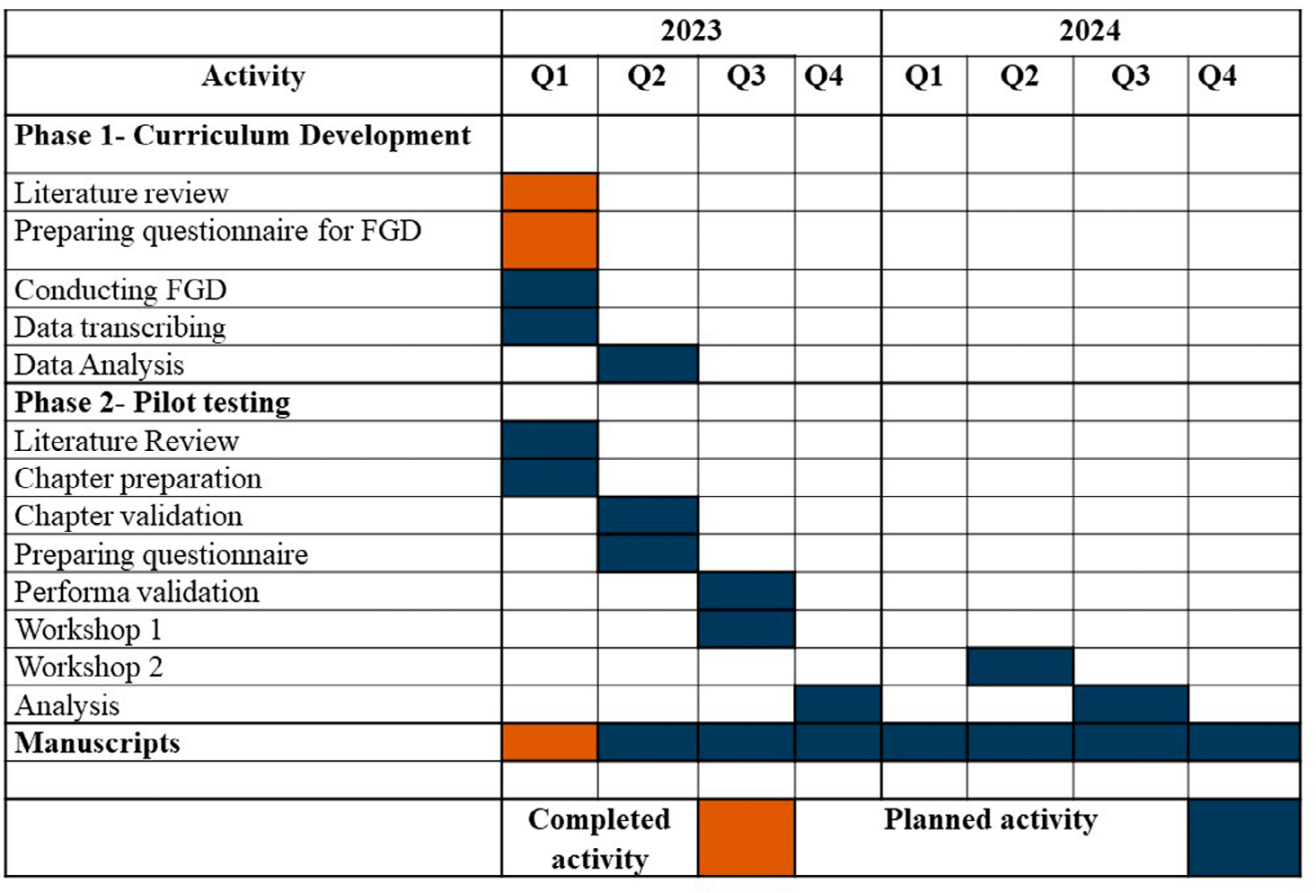
Activity plan.

Figure 2 depicts the activities and publications that will be carried out throughout the project. The project will endure for two years.

### Ethical aspects

The Institutional Ethics Committee of Kasturba Medical College and Kasturba Hospital reviewed and approved the proposal on 11 ^th^ May, 2022 (IEC1-138/2022). The protocol has been registered to the Clinical Trial Registry-India. Permission has been obtained from the local government authorities concerned, and written informed consent has been taken from the participants in the study. The data relating to the participants will be kept confidential and used, anonymously for this study only. Codes will be used for each participant.

### Potential impact of the proposed research

The curriculum developed using the data given by the participants will positively influence how LGBTQIA+ individuals are being cared for by healthcare providers. A timely contribution to discussions concerning the function of professional educational interventions is made by this project, which evaluates the impact of educational curricula and training for healthcare students and professionals on LGBTQIA+ healthcare issues. A policy document will be made at the end of the study by highlighting the study’s implications and will be disseminated among the ministries and other regulatory bodies of health and education.

## Data Availability

No underlying data are associated with this article.

## References

[ref1] Agarwal A Thiyam A : Healthcare, culture & curriculum: Addressing the need for LGBT+ inclusive medical education in India. *Lancet Reg. Health Southeast Asia.* 2022;8:100085. 10.1016/j.lansea.2022.100085 37384136 PMC10305984

[ref2] Alhanachi S Meijer LAL de Severiens SE : Improving culturally responsive teaching through professional learning communities: A qualitative study in Dutch pre-vocational schools. *Int. J. Educ. Res.* 2021;105:101698. 10.1016/j.ijer.2020.101698

[ref3] Braun V Clarke V : To saturate or not to saturate? Questioning data saturation as a useful concept for thematic analysis and sample-size rationales. *Qual. Res. Sport Exerc. Health.* 2021;13(2):201–216. 10.1080/2159676X.2019.1704846

[ref4] Chinchilla M Arcaya MC : Using Health Impact Assessment as an interdisciplinary teaching tool. *Int. J. Environ. Res. Public Health.* 2017;14(7):744. 10.3390/ijerph14070744 28698462 PMC5551182

[ref5] Cui L : Out queer teachers as role models for queer students? Implications drawn from closeted gay academics’ experiences in China. *Int. J. Educ. Res.* 2023;117:102137. 10.1016/j.ijer.2023.102137

[ref6] Gegenfurtner A : Pre-service teachers’ attitudes toward transgender students: Associations with social contact, religiosity, political preference, sexual orientation, and teacher gender. *Int. J. Educ. Res.* 2021;110:101887. 10.1016/j.ijer.2021.101887

[ref7] Grabovac I Abramović M Komlenović G : Attitudes towards and knowledge about homosexuality among medical students in Zagreb. *Coll. Antropol.* 2014;38(1):39–45. 24851595

[ref8] Hafford-Letchfield T Pezzella A Cole L : Transgender students in post-compulsory education: A systematic review. *Int. J. Educ. Res.* 2017;86:1–12. 10.1016/j.ijer.2017.08.004

[ref9] Hennink MM Kaiser BN Weber MB : What influences saturation? Estimating sample sizes in focus group research. *Qual. Health Res.* 2019;29(10):1483–1496. 10.1177/1049732318821692 30628545 PMC6635912

[ref10] Kannisto T : Trans*kinship, children’s rights, and the school. *Int. J. Educ. Res.* 2019;94:183–192. 10.1016/j.ijer.2019.01.003

[ref11] Kar A Mukherjee S Ventriglio A : Attitude of Indian medical students towards homosexuality. *East Asian Arch. Psychiatr.* 2018;28(2):59–63. 10.12809/eaap181728 29921742

[ref12] Kaufman MR Cornish F Zimmerman RS : Health behavior change models for HIV prevention and AIDS care: Practical recommendations for a multi-level approach. *J. Acquir. Immune Defic. Syndr.* 2014;66 Suppl 3(SUPPL.3):S250–S258. 10.1097/QAI.0000000000000236 25007194 PMC4536982

[ref13] Keuroghlian AS Ard KL Makadon HJ : Advancing health equity for lesbian, gay, bisexual and transgender (LGBT) people through sexual health education and LGBT-affirming health care environments. *Sex. Health.* 2017;14(1):119–122. 10.1071/SH16145 28160786

[ref29] Kitzinger J : Qualitative research. Introducing focus groups. BMJ (Clinical Research Ed.). 1995;311(7000):299–302. 10.1136/bmj.311.7000.299 7633241 PMC2550365

[ref14] Kyngäs H Mikkonen K Kääriäinen M : *The application of content analysis in nursing science research.* Springer Nature;2019.

[ref15] LaVaccare S Diamant AL Friedman J : Healthcare Experiences of Underrepresented Lesbian and Bisexual Women: A Focus Group Qualitative Study [published correction appears in Health Equity. 2018 Sep 20;2(1):233]. *Health Equity.* 2018;2(1):131–138. Published 2018 Jul 1. 10.1089/heq.2017.0041 30283859 PMC6071790

[ref16] Lundin M : Building a framework to study the hetero norm in praxis. *Int. J. Educ. Res.* 2011;50(5):301–306. 10.1016/j.ijer.2011.10.002

[ref17] Macapagal K Bhatia R Greene GJ : Differences in Healthcare Access, Use, and Experiences Within a Community Sample of Racially Diverse Lesbian, Gay, Bisexual, Transgender, and Questioning Emerging Adults. *LGBT health.* 2016;3(6):434–442. 10.1089/lgbt.2015.0124 27726496 PMC5165667

[ref18] MacKenzie A Suissa J Robertson M : ‘From the margins’: Exploring the marginalisation, exclusion and oppression of overlooked groups in philosophy of education. *Int. J. Educ. Res.* 2020;99:101504. 10.1016/j.ijer.2019.101504

[ref19] Magnus CD Dr. Lundin M Dr. : Challenging norms: University students’ views on heteronormativity as a matter of diversity and inclusion in initial teacher education. *Int. J. Educ. Res.* 2016;79:76–85. 10.1016/j.ijer.2016.06.006

[ref20] McCann E Brown M : The inclusion of LGBT+ health issues within undergraduate healthcare education and professional training programmes: A systematic review. *Nurse Educ. Today.* 2018;64:204–214. 10.1016/j.nedt.2018.02.028 29510349

[ref30] Mosack KE Brouwer AM Petroll AE : Sexual Identity, Identity Disclosure, and Health Care Experiences: Is There Evidence for Differential Homophobia in Primary Care Practice? Women’s Health Issues. 2013;23(6):e341–e346. 10.1016/j.whi.2013.07.004 24183408 PMC4141482

[ref21] Parker A Bhugra D : Attitudes of British medical students towards male homosexuality. *Sex. Relatsh. Ther.* 2000;15(2):141–149. 10.1080/14681990050010736

[ref22] Renjith V Yesodharan R Noronha J : Qualitative methods in health care research. *Int. J. Prev. Med.* 2021;12(1):IJPVM_321_19. 10.4103/ijpvm PMC8106287 34084317

[ref23] Sanchez NF Rabatin J Sanchez JP : Medical students’ ability to care for lesbian, gay, bisexual, and transgendered patients. *Fam. Med.* 2006;38(1):21–27. 16378255

[ref24] Sequeira GM Chakraborti C Panunti BA : Integrating lesbian, gay, bisexual, and transgender (LGBT) content into undergraduate medical school curricula: A qualitative study. *Ochsner J.* 2012;12(4):379–382. 23267268 PMC3527869

[ref25] Van Eeuwijk P Angehrn Z : How to … Conduct a Focus Group Discussion (FGD). *Methodological Manual.* 2017.

[ref26] Wahlen R Bize R Wang J : Medical students’ knowledge of and attitudes towards LGBT people and their health care needs: Impact of a lecture on LGBT health. *PLoS One.* 2020;15(7 July):e0234743–e0234713. 10.1371/journal.pone.0234743 32609754 PMC7329058

[ref27] Walker K Arbour M Waryold J : Educational Strategies to Help Students Provide Respectful Sexual and Reproductive Health Care for Lesbian, Gay, Bisexual, and Transgender Persons. *J. Midwifery Womens Health.* 2016;61(6):737–743. 10.1111/jmwh.12506 27783889

[ref28] Yang HC : Teaching lgbt+ health and gender education to future doctors: Implementation of case-based teaching. *Int. J. Environ. Res. Public Health.* 2021;18(16). 10.3390/ijerph18168429 34444177 PMC8394775

