# Supplementary material for: Developing a module on the care of LGBTQIA+ individuals for health professionals: Research protocol
Source: F1000Res. 2024 Jul 23;12:1437. Originally published 2023 Nov 6. [Version 2] doi: 10.12688/f1000research.140518.2 (PMC11283630; doi:10.12688/f1000research.140518.2)
Supplement: Peer review history

# Reviewer report: Reviewer response for version 2

**DOI:** 10.5256/f1000research.168961.r306059

**Published:** 2024-07-27

**Authors:** Dr shubha Jayaram

**Affiliations:** 1 Mysore Medical College and Research Institute, Mysore-570001, Karnataka, India

The authors have incorporated the changes suggested , which has improvised the previous version of the article.

Is the study design appropriate for the research question?

Partly

Is the rationale for, and objectives of, the study clearly described?

Partly

Are sufficient details of the methods provided to allow replication by others?

Partly

Are the datasets clearly presented in a useable and accessible format?

Not applicable

Reviewer Expertise:

My areas of research  are Medical Education, Diabetes Mellitus , Thanatochemistry, Global Burden of diseases,

I confirm that I have read this submission and believe that I have an appropriate level of expertise to confirm that it is of an acceptable scientific standard.

# Reviewer report: Reviewer response for version 2

**DOI:** 10.5256/f1000research.168961.r306060

**Published:** 2024-07-23

**Authors:** Vimala Ramoo

**Affiliations:** 1 Universiti Malaya, Kuala Lumpur, Federal Territory of Kuala Lumpur, Malaysia

I have no further comments.

Is the study design appropriate for the research question?

Yes

Is the rationale for, and objectives of, the study clearly described?

Yes

Are sufficient details of the methods provided to allow replication by others?

Partly

Are the datasets clearly presented in a useable and accessible format?

Not applicable

Reviewer Expertise:

Nursing education, Critical care nursing and Nursing Management and Leadership

I confirm that I have read this submission and believe that I have an appropriate level of expertise to confirm that it is of an acceptable scientific standard.

# Reviewer report: Reviewer response for version 1

**DOI:** 10.5256/f1000research.153885.r221847

**Published:** 2023-12-15

**Authors:** Dr shubha Jayaram

**Affiliations:** 1 Mysore Medical College and Research Institute, Mysore-570001, Karnataka, India

Thank you for your interest and intent in publishing this article titled, 

 “Development, implementation, and testing of LGBTQIA+ care curriculum for health science professionals: Research protocol”.  I appreciate the sincere efforts put together for the preparation of this manuscript .The topic is very relevant and the need of the hour. The following points should be addressed and the manuscript revised.

1. Title:  Title should be suitably modified to replace the word “Curriculum “  , Curriculum is a broad terminology which is defined as follows :

“Curriculum is what is taught in a given course or subject. Curriculum refers to an interactive system of instruction and learning with specific goals, contents, strategies, measurement, and resources”.  The study is a research protocol for fulfilling the identified gaps  in the health science curriculum to provide adequate care for LGBTQIA +  subjects .Therefore I suggest that the word curriculum  be modified as “ Development, implementation, and testing of a module for LGBTQIA+ care to be integrated in the curriculum for health science professionals: Research protocol" .

 2.Abstract : 

- A. “Additionally, healthcare professionals also find it difficult to care for LGBTQIA+ as very little is studied or heard about management.” This can be rephrased as “Additionally, healthcare professionals also find it difficult to care for LGBTQIA+ as they are not adequately trained regarding the management of these subjects”.
- B. Study outcome should be modified: to include   policy changing practices, not just improvement of knowledge.
- C. Keywords: Rationale for including SDG 3 and SDG -10? Sustainable Development Goal? As keywords as they nowhere appear in the manuscript and not discussed in the context, needs to be justified.

3.Page 3: Background: “Minority stress”    to be detailed in the context of health care education, the term QIA + in LGBTQIA+ is not explained.

 4.Page 3: Background: April 2014: Reference to be provided.

 5.Page 3: Background: “Although the Delhi High court……………….”, This sentence to be modified for clarity.

 6.Page 3: Background: Delhi High Court 2009:  –reference needed.

 7.Page 4:  para -1:- “Hetero sexist attitudes may result in Inaccurate Risk Assessment”  This sentence to be justified and rewritten for clarity .

 8.Page 4; para 2: Health institutions should make room for individuals –Needs to be rephrased.

 9.Page 5 : Research Plan :- points b and c ,

- b. The present health science curriculum does not have a specific curriculum that deals with the management of LGBTQIA+ health problems.
- c. There is a lack of structured guidelines for managing the health conditions of LGBTQIA+ people.   These points should be highlighted and discussed adequately as the need for the study.

10.Introduction to be rewritten, as it is not aligned with the objectives of the study. Research question is not aligned with the title of the topic and study objectives should be provided. What are ‘The gaps’ in the present curriculum / Gaps and lack of competency of medical professionals in communicating with and management of LGBTQIA+ subjects , whether it should be a part of the AETCOM modules of the present CBME (Competency Based Medical Education ) medical curriculum should be discussed in the background section.

 11.Primary Research Question and Research Objectives are not clear.

 12.In alignment with the research question, assessment of knowledge about LGBTQIA+ Care has to be included in the title.

 13.Based on the previously published literature related to the needs of LGBTQIA+, it is assumed that……….:    has to be rephrased and modified.

 14.A, B, C, points in the methodology have to be described and highlighted in the background section.

 15.Phase -1 Flow chart diagram should include obtaining Institutional Ethics Committee clearance and taking consent from the subjects.

 16.Methodology: Study design: Type of study design should be described; whether qualitative, quantitative, or Mixed methods study?

 17.Page 6: Methodology: For Focussed Group Discussions (FGD) initials of principal investigator and moderator to be mentioned.

 18.Page 6: Methodology: Inclusion criteria for FGD participants to be detailed, How were they approached? Sample size determination of participants? Criteria for inclusion?, Justification for  age group of the LGBTQIA +subjects participating in FGD, Who are all the participants of FGD apart from LGBTQIA+ subjects to be clarified; whether a separate FGD would be held for  faculty of health care professionals or whether they were also a part of the first FGD needs to be clarified and justified.

 19.Page 6: Methodology: How the recording of the non-verbal communication was done during FGD to be described.

 20.It is mentioned that the participants of the FGD will be compensated? Details required.

 21.Box -1: FGD questions: Development of FGD lead questions, details required as to how the questions of FGD were validated? And whether any formal FGD interview guide was used needs to be described.

 22.Page 4: Methodology: It is mentioned “To achieve this secondary research question, a structured knowledge-based questionnaire on LGBTQIA+ care will be administered to the health science students and faculty to assess their knowledge about LGBTQIA+ care.”   This structured questionnaire should be provided in the appendix/as annexure section. Whether Validity and Reliability of the questionnaire was tested, CVI and CVR were calculated;, whether I-CVI (Item level CVI )/ S-CVI (Scale level CVI ) were also calculated ? Validation process should be elaborated.

 23.Page 7: Phase -11: Inclusion criteria for phase -2, age group of the health care students participating in the study to be given.

 24.Page 8: How the Assessment of primary and secondary outcomes would be done; to be described.

Is the study design appropriate for the research question?

Partly

Is the rationale for, and objectives of, the study clearly described?

Partly

Are sufficient details of the methods provided to allow replication by others?

Partly

Are the datasets clearly presented in a useable and accessible format?

Not applicable

Reviewer Expertise:

My areas of research  are Medical Education, Diabetes Mellitus , Thanatochemistry, Global Burden of diseases,

I confirm that I have read this submission and believe that I have an appropriate level of expertise to confirm that it is of an acceptable scientific standard, however I have significant reservations, as outlined above.

# Response

**Published:** 2024-07-09

**Authors:** RENJULAL YESODHARAN

**Affiliations:** Manipal Academy of Higher Education, Manipal, Karnataka, India

Thank you for reviewing the manuscript. Your expert suggestions are incorporated in version 2 of the manuscript. The title of the manuscript is changed, and the "curriculum" is changed to "module". The keywords of the study have been changed as per SDG3 and SDG10. As per your suggestion, 'minority stress is explained in the text. The details you asked about FGD will be published separately as an outcome paper; hence, it is not incorporated in this manuscript. The suggestion you mentioned will be considered while disseminating the findings of FGD. As per your suggestion, we have included more explanations for assessing the primary and secondary objectives.

# Reviewer report: Reviewer response for version 1

**DOI:** 10.5256/f1000research.153885.r221846

**Published:** 2023-12-15

**Authors:** Vimala Ramoo

**Affiliations:** 1 Universiti Malaya, Kuala Lumpur, Federal Territory of Kuala Lumpur, Malaysia

**Feedback to authors**

 Dear authors, thank you for the opportunity to read and review this interesting research protocol.

 My suggestions/comments to further enhance the paper are:

**Title:**

 The title effectively conveys the primary focus and purpose of the study. It clearly indicates that the research aims to develop, implement, and evaluate a curriculum for health science professionals regarding LGBTQIA+ care. It's specific and informative, which is essential for attracting the right audience and communicating the study's objectives. Overall, the title is well-constructed and aligns with the research's objectives.

**Scope of the Study:**

 The scope of the study appears to be appropriately defined within the title and is further elaborated upon in the abstract and introduction sections of the research protocol. It includes three key phases: curriculum development, implementation, and testing, with the overarching goal of addressing healthcare disparities and enhancing LGBTQIA+ healthcare education for health science professionals.

 However, to further clarify the scope:

 In the introduction section, consider briefly mentioning the specific healthcare disciplines or professions within "health science professionals" that the curriculum is intended for (e.g., physicians, nurses, social workers) to provide a more precise context. I noted only the need for medical students and faculties is highlighted in the text. The importance for nurses and other healthcare professionals is not emphasized. Define who the healthcare providers are and use a consistent term either as healthcare providers or as healthcare professionals (healthcare science student mentioned in Figure 1). Avoid using them interchangeably, as this may confuse readers. Also, consider where students can be considered as providers or professionals.

 It is noted also the authors had mentioned assessing knowledge of LGBTQIA+ health needs and sometimes knowledge of LGBTQIA+ care – please be consistent.

**Research question**

 The research question requires minor refinement of the sentence structure, for example: How does an LGBTQIA+ curriculum impact healthcare providers' knowledge of LGBTQIA+ care?

 However, in the study the authors plan to assess the "how" using a quantitative approach, I would suggest rephrasing to:

 "To what extent does an LGBTQIA+ curriculum quantitatively improve healthcare providers’ knowledge of LGBTQIA+ care?"

 Suggestion for the secondary research question as this will be assessed using a pre-post approach:

 To assess the effectiveness of the curriculum in improving healthcare professionals’ knowledge of LGBTQIA+ care.

**Phase 1 – Development of curriculum**

 The curriculum development process should include a strong focus on cultural competence and sensitivity. This includes not only understanding healthcare needs but also addressing the unique cultural and ethical aspects of caring for LGBTQIA+ individuals.

 I would suggest that in addition to FGD and literature review, the authors should consider incorporating insights and expertise from healthcare professionals experienced in LGBTQIA+ healthcare. Experts can provide valuable guidance on best practices and ensure that the curriculum aligns with clinical reality.

 The content of the expert validation of the curriculum requires further elaboration in terms of the panel's expertise, the number of experts involved and the specific analysis process to be carried out. Future details on these aspects would be valuable to ensure transparency and accuracy in the validation process.

 I recommend determining:

- The composition of the expert panel, including the diversity of expertise among panel members (e.g., LGBTQIA+ healthcare, medical education, curriculum development).
- The total number of experts who will participate in the validation process.
- A clear description of the analysis process, indicating the criteria and guidelines against which the curriculum will be evaluated.
- How experts will provide feedback and recommendations and whether there will be a formalized assessment or evaluation system for assessing curriculum components.

FGD participants:

 While the authors mention recruiting a representative number of self-identified LGBTQIA+ individuals, consider expanding efforts to ensure diversity within the sample. Mention whether they will actively seek participants from diverse backgrounds, age groups, and gender identities to capture a comprehensive range of perspectives and experiences.

 The rationale for selecting a sample size of 10-15 participants is well-explained. The authors highlighted the importance of depth in the Focus Group Discussion (FGD) and subsequent analysis. It would be helpful to mention whether this sample size is consistent with previous research or guidelines in the field to further support the appropriateness of the sample size.

 Exclusion criteria should not be the opposite of inclusion criteria, for example: “Any person who is not belonging to the LGBTQIA+ community” is not required.

**Phase II – Pilot study**

  

- Insufficient information has been provided regarding the measurement instrument, and the questionnaire on knowledge. It is essential to include comprehensive details regarding the development or adaptation of the instrument, its content, the validation process, and reliability testing.
- The time interval between the pre-and post-test and its rationale.

Figure 1 needs a more detailed description of the process.

 Figure 2 – not clear of the activity in Workshop I.

**Discussions**

 The discussion touches on key points related to previous research and the potential impact of the curriculum. However, further elaboration, interpretation, and explicit connections between the current study and previous research would enhance the overall clarity and depth of the discussion. It would be advantageous to explicitly state how the present study seeks to build on or contribute to existing knowledge in this area. This will help readers understand the unique contributions of your research.

 All the best

Is the study design appropriate for the research question?

Yes

Is the rationale for, and objectives of, the study clearly described?

Yes

Are sufficient details of the methods provided to allow replication by others?

Partly

Are the datasets clearly presented in a useable and accessible format?

Not applicable

Reviewer Expertise:

Nursing education, Critical care nursing and Nursing Management and Leadership

I confirm that I have read this submission and believe that I have an appropriate level of expertise to confirm that it is of an acceptable scientific standard, however I have significant reservations, as outlined above.

# Response

**Published:** 2024-07-09

**Authors:** RENJULAL YESODHARAN

**Affiliations:** Manipal Academy of Higher Education, Manipal, Karnataka, India

Thank you for reviewing the manuscript. We appreciate the suggestions given by you. 

 As per your suggestions, the term ‘Medical students’ was changed to health care professionals. The research questions were modified based on the suggestions. Modifications in the figures were undertaken to accommodate these changes. We consistently used terminologies throughout the text, as per your suggestion. A few terminologies are replaced with more technical ones. Regarding the validation of the m content module, the authors have already included experienced healthcare professionals for their suggestions. The experts validate the content of the curriculum. We have also modified the discussions.
